# Hippocampal Subfield Volumes in Major Depressive Disorder Adolescents with a History of Suicide Attempt

**DOI:** 10.1155/2021/5524846

**Published:** 2021-04-12

**Authors:** Qi Zhang, Su Hong, Jun Cao, Yi Zhou, Xiaoming Xu, Ming Ai, Li Kuang

**Affiliations:** Department of Psychiatry, The First Affiliated Hospital of Chongqing Medical University, No. 1 Youyi Road, Yuzhong District, Chongqing 400016, China

## Abstract

Suicidal behavior is a leading cause of death and often commences during adolescence/young adulthood (15~29 years old). The hippocampus, which consists of multiple functionally specialized subfields, may contribute to the pathophysiology of depression and suicidal behavior. We aimed to investigate the differences of hippocampal subfield volume between major depressive disorder (MDD) patients with and without suicide attempts and healthy controls in adolescents and young adults. A total of 40 MDD suicide attempters (MDD+SA), 27 MDD patients without suicide attempt (MDD-SA), and 37 healthy controls (HC) were recruited. High-resolution T1 MRI images were analyzed with the automated hippocampal substructure module in FreeSurfer 6.0. Volume differences among the groups were analyzed by a generalized linear model controlling for intracranial cavity volume (ICV). The relationship between hippocampal subfield volumes and clinical characteristics (HAM-D and SSI scores) was assessed using two-tailed partial correlation controlling for ICV in MDD+SA and MDD-SA. We found that MDD-SA had significantly smaller bilateral hippocampal fissure volume than HC and MDD+SA. No significant correlation was observed between hippocampal subfield volume and clinical characteristics (HAM-D and SSI scores) in MDD+SA and MDD-SA. Adolescent/young adult suicide attempters with MDD suicide attempters have larger bilateral hippocampal fissures than depressed patients without suicide attempts, independently from clinical characteristics. Within the heterogeneous syndrome of major depressive disorder that holds a risk for suicidality for subgroups, hippocampal morphology may help to explain or possibly predict such risk, yet longitudinal and functional studies are needed for understanding the biological mechanisms underlying.

## 1. Introduction

Major depressive disorder (MDD) is a prevalent, highly debilitating disease, with a 2%-12% lifetime suicide risk [1, 2]. Although it was originally regarded as an extreme outcome of MDD and then as a potential confounding factor in studies of mood disorder neurobiology, suicide is increasingly thought to have its own unique neurobiological mechanisms [3, 4]. Despite multiple common genetic and environmental risk contributors, only a small proportion of MDD patients confronted with acute stressors will commit suicide. In particular, suicide is a leading cause of death during adolescence/young adulthood (15~29 years old) [1, 2] when the frontal lobe and limbic system implicated in suicidal behavior are continuing to develop [5, 6]. Therefore, understanding the neurobiology and circuits of suicidality in MDD is crucial for alleviating the impact of the devastating illness on public health.

Studies indicate that synaptic plasticity impairment in specific areas of the CNS, particularly the hippocampus, has been linked to mood disorders that usually occur during adolescence or early adulthood, which have major cognitive and emotional symptoms [7, 8]. Substantial evidence suggests that the hippocampus plays a crucial role in memory formation, and memory dysfunction has been linked to suicide attempts [9–11]. The hippocampus is also involved in other complex behaviors, such as regulating emotional responses, executive function, sensorimotor integration, and goal-directed activity, the alterations of which may be associated with suicide attempts [12]. Data on hippocampal volume related to suicide attempts in adolescents/young adults are scant. Convergent structural magnetic resonance imaging (sMRI) evidence in adult attempts across psychiatric disorders supports the involvement of the hippocampus [13–16]. However, the findings of hippocampal volume in suicide attempters with MDD are not consistent, showing no changes [17, 18], smaller volumes [12, 13], and even larger volume [19] in suicidal MDD patients compared to healthy controls and nonsuicidal patient controls. The inconsistent results may be related to the different medication status, disease severity, age of onset, and suicidality assessments in these studies. Additionally, because of the high neuroplasticity and variability of brain development in adolescence/young adulthood, adult neurobiological findings may not be directly applied to youth. The mechanisms that lead to distinct structural changes in the hippocampus between MDD with and without suicide attempt are still uncovered. Further variability may be due to the hippocampus consisting of multiple subfields with distinct morphology [20], by which cellular and molecular mechanisms associated with mood disorders may be localized to. Therefore, it is crucial to explore to which extent the hippocampal subfields of suicide attempters alter to better understand the role the hippocampus plays in suicidal MDD.

In this study, young MDD patients with and without suicide attempts as well as healthy controls were evaluated by automated hippocampal substructure segmentation of high-resolution T1-weighted images. This study is aimed at identifying volume alteration in the hippocampus in suicidal MDD. We hypothesized that young MDD patients with suicide attempts would demonstrate abnormalities in specific hippocampal subfields, and these abnormalities would be linked with MDD and suicidal ideation severity.

## 2. Methods

### 2.1. Participants

Participants were between 14 and 25 years old and included patients with MDD and a history of at least one suicide attempt (MDD+SA), patients with MDD and no lifetime history of suicide attempt (MDD-SA), and healthy controls (HC) with no current or history of any major DSM-IV Axis I diagnosis and no history of suicidal behavior. All subjects were Chinese Han ethnicity and right-handed, without contraindications for magnetic resonance imaging investigation. The study protocol was approved by the Ethics Committee of the First Affiliated Hospital of Chongqing Medical University. The study was performed in compliance with the Code of Ethics of the World Medical Association (Declaration of Helsinki). All participating individuals were informed of the procedures of the study. Written informed consent was obtained from participants ≥ 18 yrs, and permission from parents or legal guardians and assent from minors before the study.

MDD patients were recruited from consecutive referrals to the Department of Psychiatry, the First Affiliated Hospital of Chongqing Medical University. A suicide attempt was defined as a self-injurious act causing physical harm with some intent to die, committed within 6 months prior to magnetic resonance scanning. All patients were assessed by two experienced psychiatrists with the Structured Clinical Interview for DSM-IV Diagnosis (SCID) [21] for participants ≥ 18 yrs and the Chinese version of Kiddle-Schedule for Affective Disorders and Schizophrenia [22] if <18 yrs and met the criteria for depressive disorder. The enrolled patients were free of antidepressant medication for at least two weeks and had refrained from electroconvulsive therapy for at least six months at the time of scanning. Current mood symptoms were assessed with the 17-item Hamilton depression rating scale (HAM-D) [23]. The Beck Scale for Suicide Ideation [24] was used to assess suicide ideation severity.

Healthy controls were recruited through flyers and internet advertisements from the local community. Enrolled healthy controls were screened through the Structured Clinical Interview for DSM-IV nonpatient edition (SCID-NP), to ensure that the diagnosis of depression and other psychiatric diseases was excluded. Common exclusion criteria for all subjects were any other DSM-IV Axis I disorder, history of head trauma with residual effects, neurological disorders, substance or alcohol abuse/dependence at any time, uncontrolled major medical conditions, history of psychiatric disorders or suicide among first-degree relatives, and other clinically relevant abnormalities in the medical history or laboratory examinations.

### 2.2. MRI Data Acquisition

T1-weighted anatomical images were acquired with a 3.0-Tesla GE Sigma HDxt MRI system (General Electric Healthcare, Chicago, Illinois, USA) using a standard 8-channel head coil with the following parameters: repetition time (TR) = 24 ms, echo time (TE) = 9 ms, flip angle = 90, field of view (FOV) = 240 mm, slice thickness = 1 mm, and acquisition matrix = 256 × 256. All scans were visually reviewed by a neuroradiologist to check for incidental findings, and no obvious gross abnormalities were ruled out. One subject from the SA group was excluded for panic attack during the scan.

### 2.3. Image Processing

Subcortical reconstruction and segmentation were performed using the FreeSurfer software suite version 6.0 (http://surfer.nmr.mgh.harvard.edu). The technical details of the procedures including motion correction, intensity normalization, automated topology corrections, and automatic segmentations were described elsewhere [25–27]. The hippocampal module was used to parcellate the hippocampus subfields. Twelve distinct subfields were obtained for the hippocampus: cornu ammonis (CA1), CA2/3, CA4, molecular layer, granular cell layer of the dentate gyrus (GC-DG), hippocampus-amygdala transition area (HATA), subiculum, presubiculum, parasubiculum, fimbria, hippocampal tail, and fissure. We conducted quality control of segmentation following the ENIGMA consortium's quality assurance protocol (http://enigma.ini.usc.edu/ongoing/enigma-hippocampal-subfields). No subject was excluded due to poor subfield segmentation.

### 2.4. Statistical Analyses

Statistical analyses were conducted using SPSS for Mac (version 26.0; IBM Corp., Armonk, NY). The chi-squared tests were employed to test for group differences in categorical variables, such as gender. One-way ANOVAs were employed to evaluate differences in continuous variables, such as age and years of education. For the clinical subjects (MDD-SA and MDD+SA), a *t*-test was also conducted to test for differences in the HAM-D score and SSI score.

Differences in hippocampal subfield volumes among the three groups were analyzed using general linear model (GLM) analyses. In these and all analyses, intracranial volume (ICV) was used as a covariate, and the false discovery rate (FDR) correction was performed in multiple comparisons across the 24 subfields. FDR correction is a less conservative approach but appropriate for exploratory analysis. Bonferroni *post hoc* analysis was performed between the three groups to identify the pairwise group differences. We also made an additional *t*-test comparing MDD cases with very low SSI (the Beck Scale for Suicide Ideation total score < 10) with all the MDD+SA cases. Hippocampal subfields with significant differences among the three groups were subsequently considered regions of interest (ROIs). The receiver operating characteristic (ROC) method was used to evaluate the volume of ROIs as markers to distinguish patients with suicide attempt from depressed patients. The Youden index was used to ensure the threshold of diagnosis accuracy for ROIs.

The relationship between hippocampal subfield volume and clinical characteristics (HAM-D and SSI scores) was assessed using partial correlation controlling for ICV. The correlation analyses were performed in MDD+SA and MDD-SA. All statistical tests were two-tailed, and the significance level was set as 0.05.

## 3. Results

A total of 104 subjects were recruited (40 MDD+SA, 27 MDD-SA, and 37 HC).  Table 1 demonstrates the demographic and clinical characteristics of the sample (Table 1). There were no significant differences in age (*P* = 0.084), sex (*P* = 0.360), and education (*P* = 0.235) among the three groups, and there were no significant differences in the HAM-D score (*P* = 0.845) between MDD-SA and MDD+SA. Attempters and patient controls differed significantly in suicide ideation severity (*t* = 3.158, *P* = 0.002). The attempters had 2.86 ± 2.16 attempts on average.

**Figure 1:**
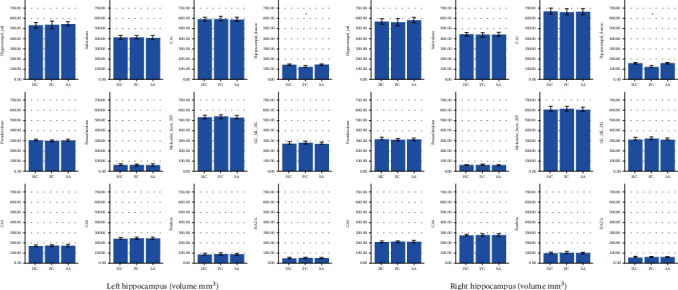
Hippocampal subfield volumes in HC, PC, and SA. ^∗^*P*_FDR_ < 0.05. Error bar indicates one standard error. Abbreviations: HC: healthy controls; PC: patient controls; SA: suicide attempters; CA: cornu ammonis; GC: granular layer; ML: molecular layer; DG: dentate gyrus; HATA: hippocampus-amygdala transition area.

Mean hippocampal subfield volumes are shown in Figure 1. There was no significant group effect found in left (*F* = 0.412; *P* = 0.663) and right (*F* = 0.066; *P* = 0.936) whole hippocampal volumes and ICV (*F* = 0.145; *P* = 0.865). Adjusted *P* values showed a significant group effect on bilateral hippocampal fissure (left: *F* = 7.900; *P*_FDR_ = 0.012; right: *F* = 9.161; *P*_FDR_ < 0.001) and bilateral fimbria (left: *F* = 3.319; *P*_FDR_ = 0.032; right: *F* = 3.108; *P*_FDR_ = 0.042). *Post hoc* pairwise comparison showed that PC had significantly smaller bilateral hippocampal fissure volume than HC and SA (Table 2). The comparison of bilateral fimbria volumes did not survive the Bonferroni *post hoc* test. The further comparison showed that MDD+SA cases had significantly larger bilateral hippocampal fissure volume (left: *P* = 0.040, right: *P* = 0.009) and smaller left fimbria (*P* = 0.023) than MDD cases with very low SSI.

**Figure 2:**
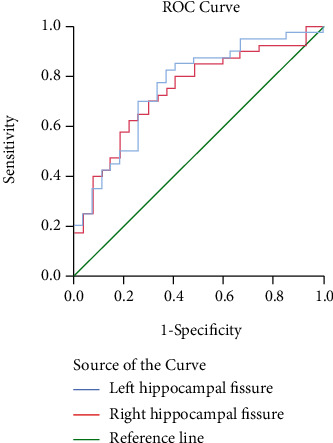
The left and right fissure volumes as discriminators of the SA and PC groups (receiver operating characteristic).

The discriminant and receiver operating characteristic analyses revealed that the left hippocampal fissure volume of 131 mm^3^ (82.5% sensitivity, 63.0% specificity) and the right hippocampal fissure volume of 149 mm^3^ (70.0% sensitivity, 70.4% specificity) allowed optimal discrimination between SA and PC (Figure 2).

No significant correlation was observed between hippocampal subfield volumes and clinical characteristics (HAM-D and SSI scores) in SA and MDD-SA (Table 3).

## 4. Discussion

Suicidal behavior is an extreme act of subjects with MDD compounded by environmental stimuli with biological vulnerability resulting from impaired brain structural plasticity [28]. Suicidal behavior has unique pathobiology which differs from MDD in general; differences have been observed in neurons, neurotransmitter receptors, glial cells, and white matter in the prefrontal cerebral cortex [15, 29]. Our findings are consistent with the notion and suggest that the biological basis for the presence of suicidal behavior in youth MDD involves hippocampal substructural changes. In this study, we observed that MDD patients with suicide attempt had larger bilateral hippocampal fissures than depressed patients without suicide attempts. Further analysis showed that hippocampal subfield volume changes were not correlated with the severity of the disorder or suicidal ideation.

Given its role in the encoding and recall of the emotional significance of events, the hippocampus may influence emotional reactions and regulatory processes. Moreover, impairment of memory has been associated with suicide attempts, implicating the involvement of the hippocampus in this process. Although hippocampal volume reductions have been observed in adult [7, 13, 14, 30, 31] and adolescent [32] attempters with MDD, our study failed to replicate these findings. Varying findings may be due to differences in imaging methodology, subject samples, and instruments to assess suicidality. With the hippocampal segmentation approach, the results showed that the hippocampus volume alteration of suicide attempter with MDD was complicated, which is combined with an increase and decrease in different hippocampal subfields simultaneously. Additionally, the reduced hippocampal volume in MDD only is observed in individuals with multiple depressive episodes and/or longer duration of the illness [31, 33–35], while in our study, most of the young MDD patients had single depressive episodes and shorter duration.

In line with our results, the hippocampus volume showed no significant difference in the MDD and MDD+SA groups in postmortem morphometric studies [15, 36]. Among the young MDD patients in our study, those with suicide attempts showed larger volumes in bilateral hippocampal fissures. The presence of enlarged hippocampal fissures may indicate abnormal neurodevelopment in the hippocampus. The fusing of bilateral fissures is normally complete during gestation; a disruption of the process could result in enlarged fissure volume [37]. The results suggested that disrupted hippocampal development before birth might be a trait and not a state of youth MDD suicide attempter. The increase of hippocampal fissure volume has been reported to highly correlate with a decrease in overall hippocampal volume, and it can serve as a radiological marker of ongoing hippocampal atrophy [38–41]. Therefore, the increased bilateral hippocampal fissure volume observed in SA may imply impending whole hippocampus atrophy. This kind of lesion has also been reported in patients with first-episode schizophrenia [42], Alzheimer's disease [43–45], and Parkinson's disease [46], which is consistent with disrupted neurodevelopment in this brain region. Longitudinal studies are needed to follow the possible secondary reduction in hippocampal volume caused by hippocampal developmental disruption that happened in early life. Bilateral hippocampal fissure volumes increase in suicide attempters compared to healthy controls which did not reach significance. Although the mechanism remains unclear, it cannot be simply assumed that SAs had similar hippocampal fissure as HCs. These hippocampal subfield volumes may better differentiate attempter status within MDD. The comparison of bilateral hippocampal fissure volumes in suicide attempters and healthy controls did not reach statistical significance. Although the mechanism remains unclear, it cannot be simply assumed that MDD+SAs had similar hippocampal fissures as HCs. These hippocampal subfield volumes may better differentiate attempter status within MDD. The ROC analysis suggested that the bilateral hippocampal fissure volumes were markers distinguishing suicidal from nonsuicidal depression with satisfied sensitivity and specificity. Our findings support the hypothesis that the pathobiology mechanism behind suicide differs from depression in general.

Although it did not survive Bonferroni correction, we observed that bilateral fimbria volume in MDD+SA decreased compared to MDD-SA and HC. As a main output structure of the polysynaptic intrahippocampal pathway (PIP), the fimbria plays a crucial role in the encoding and consolidation process of memory formation [47]. Efferent nerve fibers of the fimbria project to the limbic system and the cortex and the fibers of the fimbria fornix link the two hippocampi together. Impairment of these connections may contribute to frontolimbic network dysfunction, accounting for the emotional disturbances and cognitive deficits [48]. The significant reduction of fimbria volume was found in females with MDD [49] and bipolar patients [50]. Moreover, decreased volume of the fimbria was also shown in neurodegenerative diseases, such as essential tremor [51], Alzheimer's disease, and mild cognitive impairment patients [52–54].

Our findings are in line with previous studies [13, 55], showing that there were no significant effects of depression severity on hippocampal volume in depressed patients. To our knowledge, although numerous evidences have demonstrated the correlation between hippocampal volume alteration with mood disorders and trauma history, there was no direct association found between hippocampal volume and suicidal behavior [17, 56]. Our study is consistent with the lack of a direct relationship. One possibility is that the dysfunction and volume alteration of the hippocampus are mainly the results of childhood traumatic events, which only represent a subset of suicide attempts [7, 57].

There are several limitations that warrant discussion. The present study cannot establish causality between hippocampus subfield alternation and suicide attempt due to the cross-sectional design. Future longitudinal prospective studies are needed to follow young depressed patients to compare the alteration of the hippocampus before and after a suicide attempt and track the changes of suicidal ideation to behavior. The study did not examine the variability associated with multiple potentially relevant dimensions, such as disease duration, age of onset, and the lethality of suicide attempts, which reduces the power to interpret the findings. Additionally, of note, the present study has a wide age range covering both pubertal teenagers and young adults from 14 to 25 years old. Although age was included as a covariate, the effect of brain development would be ignored. In addition, the moderate sample size might have limited the statistical power to identify less robust effects. Thus, future studies with larger samples are warranted.

In summary, the present study provides evidence of the impact of suicidality on hippocampus subfield volume in adolescent/young adult MDD patients. Depressed suicide attempters have larger bilateral hippocampal fissures than depressed patients without suicide attempts, independently from clinical characteristics. Longitudinal studies will be helpful to understand the causal relationship between disorder progression and volumetric changes in hippocampal subfields.

## Figures and Tables

**Table 1:** Demographic and clinical information of the subjects.

	HC (*n* = 37)	PC (*n* = 27)	SA (*n* = 40)	*P*
Age (years)	20.03 ± 2.08	18.52 ± 3.11	19.60 ± 2.87	0.084
Sex				0.465
Male	15	7	13	
Female	22	20	27	
Education (years)	13.16 ± 1.54	12.33 ± 2.72	12.55 ± 1.96	0.235
HAM-D		16.96 ± 5.89	20.78 ± 5.47	0.845
SSI		12.63 ± 5.96	16.90 ± 5.04	0.002
Number of previous attempts	—	—	2.86 ± 2.16	

Abbreviations: SA: suicide attempters; PC: patient controls; HC: healthy controls; HAM-D: Hamilton depression rating score; SSI: scale for suicidal ideation.

**Table 2:** Hippocampal subfield volume difference between healthy controls, patient controls, and suicide attempters with MDD.

Subfields	HC (*n* = 37)	PC (*n* = 27)	SA (*n* = 40)	*F*	*P*	*P*_FDR_	HC vs. PC	HC vs. SA	PC vs SA
Left whole	3298.54 ± 302.50	3322.77 ± 278.58	3290.71 ± 321.39						
Left hippocampal tail	532.70 ± 77.41	536.28 ± 90.47	546.15 ± 64.26	0.426	0.654	1.000	1.000	1.000	1.000
Left subiculum	420.03 ± 41.75	422.15 ± 39.41	418.21 ± 48.58	0.197	0.822	1.000	1.000	1.000	1.000
Left CA1	598.25 ± 63.63	602.20 ± 57.48	591.35 ± 70.84	0.395	0.675	1.000	1.000	1.000	1.000
Left hippocampal fissure	147.61 ± 23.23	127.46 ± 17.03	147.34 ± 26.43	7.900	0.001^∗^	0.012^∗^	0.002	1.000	0.001
Left presubiculum	310.00 ± 34.21	302.90 ± 29.24	307.90 ± 37.79	0.293	0.747	1.000	1.000	1.000	1.000
Left parasubiculum	66.26 ± 12.10	64.68 ± 10.76	62.17 ± 9.40	1.585	0.210	1.000	1.000	0.258	0.787
Left molecular-layer-HP	538.95 ± 49.98	542.05 ± 46.33	534.51 ± 57.16	0.422	0.657	1.000	1.000	1.000	1.000
Left GC-ML-DG	278.40 ± 36.04	283.67 ± 29.72	277.50 ± 34.13	0.735	0.482	1.000	0.763	1.000	0.963
Left CA3	176.21 ± 25.92	181.02 ± 25.10	180.48 ± 27.26	0.718	0.490	1.000	0.839	1.000	1.000
Left CA4	237.83 ± 30.98	242.58 ± 25.88	237.38 ± 28.16	0.740	0.480	1.000	0.751	1.000	0.974
Left fimbria	88.00 ± 21.70	93.91 ± 16.24	84.61 ± 24.09	3.319	0.004^∗^	0.032^∗^	0.436	1.000	0.181
Left HATA	51.93 ± 8.77	51.33 ± 7.29	50.43 ± 7.38	0.344	0.709	1.000	1.000	1.000	1.000
Right whole	3427.53 ± 344.82	3412.03 ± 306.43	3427.53 ± 336.36						
Right hippocampal tail	549.32 ± 65.18	540.73 ± 81.40	562.13 ± 83.83	0.835	0.437	1.000	1.000	1.000	0.738
Right subiculum	429.82 ± 44.40	425.51 ± 45.34	427.14 ± 46.62	0.012	0.988	1.000	1.000	1.000	0.705
Right CA1	645.82 ± 74.76	631.60 ± 78.42	638.65 ± 73.12	0.129	0.879	1.000	1.000	1.000	1.000
Right hippocampal fissure	161.00 ± 20.45	140.28 ± 20.02	159.82 ± 23.88	9.161	<0.001^∗∗^	<0.001^∗∗^	0.001	1.000	0.001
Right presubiculum	297.08 ± 37.50	287.48 ± 32.00	289.91 ± 36.35	0.452	0.638	1.000	1.000	1.000	1.000
Right parasubiculum	63.76 ± 10.53	59.06 ± 9.61	60.09 ± 9.08	1.791	0.172	1.000	0.287	0.375	1.000
Right molecular-layer-HP	565.99 ± 58.58	564.33 ± 57.26	561.18 ± 58.51	0.082	0.922	0.998	1.000	1.000	1.000
Right GC-ML-DG	290.48 ± 38.07	297.93 ± 32.41	293.75 ± 34.72	0.983	0.378	1.000	0.496	1.000	1.000
Right CA3	192.22 ± 29.22	201.42 ± 27.09	199.86 ± 28.41	1.762	0.177	1.000	0.269	0.431	1.000
Right CA4	248.70 ± 31.54	256.30 ± 27.65	251.53 ± 30.18	1.238	0.295	1.000	0.356	1.000	1.000
Right fimbria	89.77 ± 22.89	95.30 ± 16.73	88.92 ± 20.14	3.108	0.007^∗^	0.042^∗^	0.459	1.000	0.342
Right HATA	54.57 ± 7.75	52.37 ± 6.20	54.37 ± 9.26	0.592	0.555	1.000	0.974	1.000	1.000

^a^Bonferroni corrected for multiple comparisons between the groups. ^∗^*P* < 0.05, ^∗∗^*P* < 0.001. Abbreviations: SA: suicide attempters; PC: patient controls; HC: healthy controls; CA: cornu ammonis; GC-DG: granule cell layer of dentate gyrus; HATA: hippocampus-amygdala transition area; FDR: false discovery rate.

**Table 3:** Correlations observed between hippocampal subfield volumes in patients of major depressive disorder (SA and PC) and clinical characteristics (HAM-D and SSI scores).

Subfields	HAM-D	SSI
Left hippocampal tail	0.085	-0.030
Left subiculum	0.135	-0.023
Left CA1	0.065	-0.126
Left hippocampal fissure	0.009	0.065
Left presubiculum	0.018	-0.068
Left parasubiculum	-0.161	-0.164
Left molecular-layer-HP	0.055	-0.116
Left GC-ML-DG	0.089	-0.068
Left CA3	0.075	-0.102
Left CA4	0.105	-0.083
Left fimbria	-0.005	0.062
Left HATA	0.116	-0.205
Right hippocampal tail	0.053	-0.062
Right subiculum	0.155	0.112
Right CA1	0.105	-0.004
Right hippocampal fissure	0.042	0.093
Right presubiculum	0.101	0.141
Right parasubiculum	0.140	-0.030
Right molecular-layer-HP	0.084	0.019
Right GC-ML-DG	0.098	0.008
Right CA3	-0.021	-0.061
Right CA4	0.113	0.031
Right fimbria	-0.034	-0.040
Right HATA	0.127	-0.051

Abbreviations: SA: suicide attempters; PC: patient controls; HAM-D: Hamilton depression rating score; SSI: scale for suicidal ideation; CA: cornu ammonis; GC-DG: granule cell layer of dentate gyrus; HATA: hippocampus-amygdala transition area.

## Data Availability

Access to data is restricted.
